# Reducing prediction volatility in the surgical workflow recognition of endoscopic pituitary surgery

**DOI:** 10.1007/s11548-022-02599-y

**Published:** 2022-04-01

**Authors:** Adrito Das, Sophia Bano, Francisco Vasconcelos, Danyal Z. Khan, Hani J Marcus, Danail Stoyanov

**Affiliations:** 1 Wellcome/EPSRC Centre for Interventional and Surgical Sciences, University College London, London, United Kingdom; 2 Department of Neurosurgery, National Hospital for Neurology and Neurosurgery, London, United Kingdom

**Keywords:** Surgical video analysis, Temporal smoothing functions

## Abstract

**Purpose:**

Workflow recognition can aid surgeons before an operation when used as a training tool, during an operation by increasing operating room efficiency, and after an operation in the completion of operation notes. Although several methods have been applied to this task, they have been tested on few surgical datasets. Therefore, their generalisability is not well tested, particularly for surgical approaches utilising smaller working spaces which are susceptible to occlusion and necessitate frequent withdrawal of the endoscope. This leads to rapidly changing predictions, which reduces the clinical confidence of the methods, and hence limits their suitability for clinical translation.

**Methods:**

Firstly, the optimal neural network is found using established methods, using endoscopic pituitary surgery as an exemplar. Then, prediction volatility is formally defined as a new evaluation metric as a proxy for uncertainty, and two temporal smoothing functions are created. The first (modal, $M_n$) mode-averages over the previous *n* predictions, and the second (threshold, $T_n$) ensures a class is only changed after being continuously predicted for *n* predictions. Both functions are independently applied to the predictions of the optimal network.

**Results:**

The methods are evaluated on a 50-video dataset using fivefold cross-validation, and the optimised evaluation metric is weighted-$F_1$ score. The optimal model is ResNet-50+LSTM achieving 0.84 in 3-phase classification and 0.74 in 7-step classification. Applying threshold smoothing further improves these results, achieving 0.86 in 3-phase classification, and 0.75 in 7-step classification, while also drastically reducing the prediction volatility.

**Conclusion:**

The results confirm the established methods generalise to endoscopic pituitary surgery, and show simple temporal smoothing not only reduces prediction volatility, but actively improves performance.

## Introduction

Surgical workflow analysis is the breakdown of a surgical procedure by splitting it into several well-defined phases, which are then further broken down into a series of well-defined steps [1]. It has been shown to reduce operative time by increase operating room efficiency, be an objective measure in assessing surgical skill, as well as be useful in training surgeons [2]. The automated recognition of surgical phases and surgical steps enables context-aware systems to aid clinicians both intra-operatively and post-operatively [2]. Intra-operative (online) recognition allows for the detection of real-time problems, such as unexpected variations and rare cases, and help less experienced surgeons with decision making [2]. It can also be used to prompt the wider operating room team (e.g. anaesthetists and theatre nurses) throughout the procedure, such as when a new tool is required, in order to maximise operating room throughput [3]. Post-operative (offline) recognition reduces the time and effort needed to complete operation notes and can be used by clinicians to both review operations and assess skills at a later date [2].

Although historically several machine learning models have been used in automated workflow recognition, in recent years the more successful approaches have been with artificial neural networks (ANN) [3, 4]. These involve using a convolution neural network (CNN) as a baseline network, due to their proficiency in image classification, and improving the results with methods that utilise the temporal data present in the videos. Most commonly, a specific type of CNN, called a residual neural network, is used as this baseline; however, the temporal methods have less of a consensus. They can be divided into two categories: (i) utilising the temporal data directly via a neural network, and (ii) utilising the sequential nature of the CNN output and using mathematical techniques to account for the misclassifications. In (i), recurrent neural networks (RNN) are most common [3, 4], in particular long short-term memory networks (LSTM) [5], although temporal convolution networks (TCN) [6, 7] have also been shown to be effective. In (ii), hidden Markov models (HMM) are most often used [3, 8], although prior knowledge inference (PKI) [9], and temporal smoothing functions (TSF) [10, 11] have also been shown to improve performance.

**Fig. 1:**
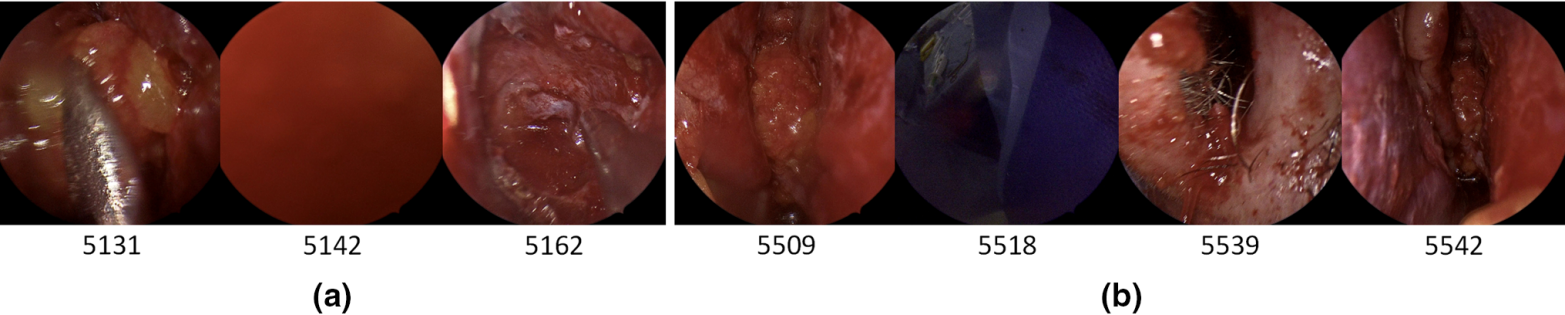
Example of uninformative images that could belong to any step, sandwiched between informative images. All images are from the same step and video, and under each image is the number of seconds into the video the image is taken from. (a) Represents a series of blurry images (e.g. 5142). (b) Represents images of the operating room (e.g. 5518), followed by images of nasal entry (e.g. 5539)

**Fig. 2:**
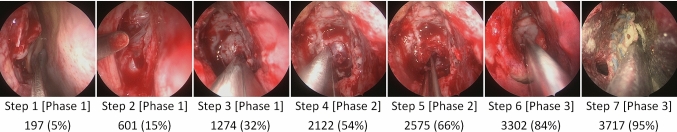
Surgical phases and steps example images from a single video. Under each image is the number of seconds into the video the image is taken from, and in brackets the percentage of video completion this represents. Note the differences in the images, particular the instruments, which the CNN will learn the features of to discriminate between the classes

The development of these methods has been limited to a handful of surgeries [3, 12]; primarily laparoscopic cholecystectomy [5, 8–11], with a few studies in cataract [13] and laparoscopic gastric bypass surgery [7], as summarised by Table 1 and Table 2 in supplementary material. Therefore, the established methods are potentially overfitting to cholecystectomy, and it is important to check whether they are generalisable to other surgeries. As an exemplar, endoscopic pituitary surgery represents a unique computer vision challenge as the working space is far smaller, and the image is therefore susceptible to occlusion from instruments or bleeding, often necessitating in the frequent withdrawal of the endoscope to adjust its position or to clean it. This results in short periods of uninformative images as displayed in Fig. 1. As will be shown (e.g. Fig. 4), the established methods are not suited for this environment, and lead to volatile predictions, where the predicted surgical phase or step rapidly changes from one second to the next. Moreover, pituitary surgery has three surgical phases with few phase transitions per surgery [1, 14], compared to the many more phases and transitions in other surgeries where workflow recognition has been automated [3]. This means reducing the prediction volatility in pituitary surgery will comparatively have a greater impact due to its intrinsic workflow characteristics. Since the eventual goal of automated workflow recognition is to be used within a clinical setting, having highly volatile predictions would detract from many of the benefits, and clinicians will question the validity of the output [15]. Specifically, the poor contextualisation of uncertainty in ANNs and the “black box” nature of ANNs have been identified as key barriers for the use of these models within brain tumour surgery [15]. Therefore, for pituitary surgery, using volatility as a proxy for uncertainty is useful for explainability, and utilising TSFs to reduce this volatility while retaining performance will increase clinical confidence.

The precedence laid out in the previous studies provides good evidence that similar methods are applicable to other automated surgical workflow recognition tasks. Therefore, the contribution in this study is twofold:

(1) A detailed comparison of established automated operative workflow recognition methods to a new surgery, demonstrating their successes or failures, highlighting that this 50-video large dataset is unique and is labelled with both surgical phases and surgical steps.

(2) Two simple temporal smoothing functions, both of which increase performance while reducing the prediction volatility.

## Methods

### Pituitary surgery dataset

**Fig. 3:**
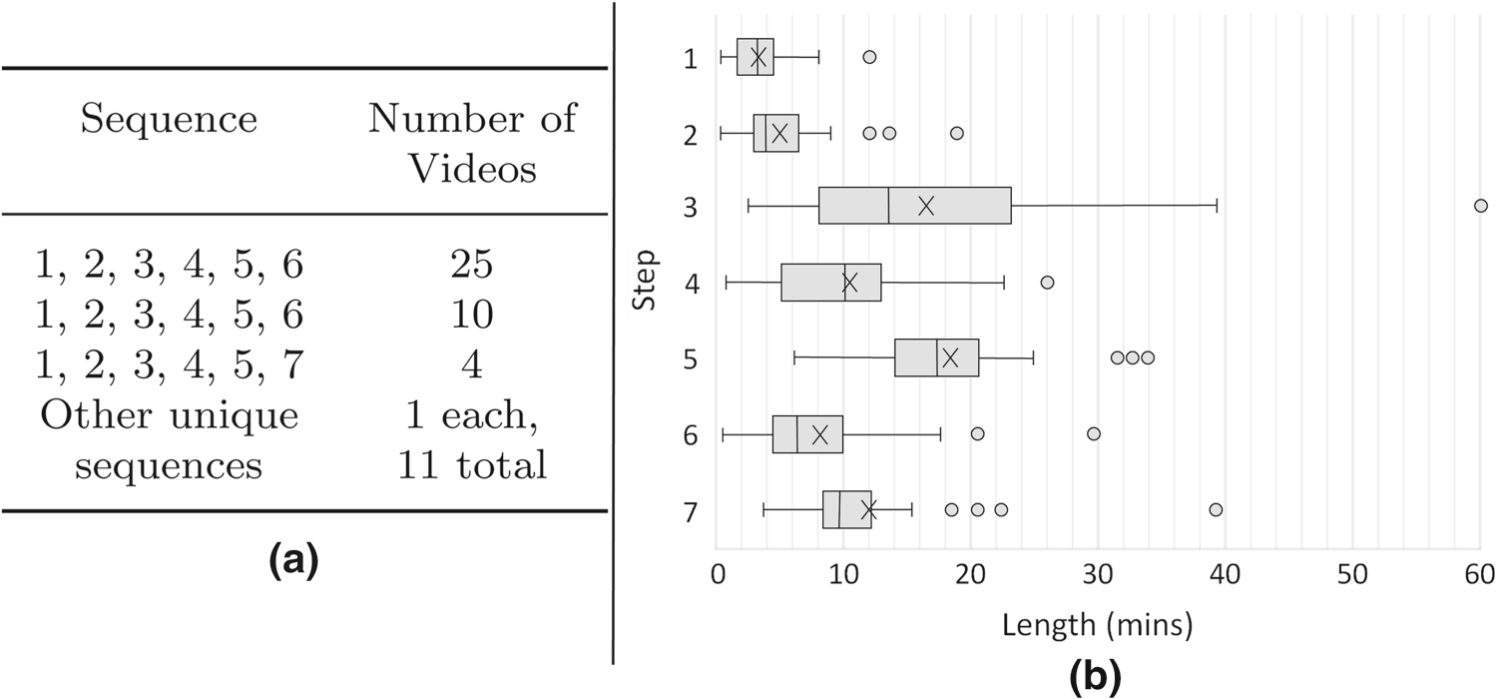
**(a)** Step sequence variation.** (b)** Step length distribution. The inter-quartile ranges are 25%, excluding the outliers, which are represented as dots. Each cross represents the mean value for that step. Across all steps, the mean is 74 mins, and the median is 67 mins with a 56–85 mins inter-quartile range

The pituitary surgery was divided into 3 surgical phases and 7 surgical steps as defined clinically in [14], with example images displayed in Fig. 2. Fifty videos are available in this dataset; 45 are complete and 5 have minor losses. Fig. 3 shows the variation in the step sequencing, and the distribution of the step lengths.

### Spatial recognition

This paper contains three incremental improvements in methodology: (i) spatial recognition, (ii) spatial-temporal recognition, and (iii) temporal smoothing. The evaluation metric to be maximised is weighted-$F_1$ score, the weighted mean of $F_1$ scores across all classifications. This has been chosen as it accounts class imbalance, and ensures a high accuracy while also safeguarding against small precision or recall. A random fivefold cross-validation split (40-training to 10-validation) was used to ensure generalisability of results, and all results are judged on the averaged online performance on the validation dataset. All 50 videos have at frame rate of 25 frames per second, with resolutions varying from 720p–2160p, and stored as mp4 files. Each video was converted to jpeg images at 1 frame per second, with the resolution converted to 720p for consistency and to improve on computational time. The code is written in Python 3.8 using PyTorch 1.8.1, and will be publicly available. Neural networks are run on a NVIDIA Tesla V100 Tensor Core 32 GB GPU using CUDA 11.2.

A multitude of CNNs were trailed to find the optimal spatial method. All networks are pre-trained on ImageNet, and run with batch size 8 for 8 epochs. The optimiser used is stochastic gradient descent (SGD) with learning rate 0.001 and momentum 0.9, and the Max activation function is used for the classification layer. The training dataset images were randomly augmented before all images are resized to 224$\times$224 pixels and colour normalised to match the ImageNet dataset. To prevent class imbalance, all classes in the training datasets were downsampled to the class with the fewest number of images. For phases, this was phase 3, with $\sim$35000 images per phase, and for steps this was step 1 with $\sim$7500 images per step, depending on the cross-validation split. This means training images were sampled, on average, every 3 seconds for both phases and steps. No such limits were applied to the valuation datasets.

As seen in Table  1, excluding AlexNet, the performances of the networks are almost identical. This suggests the networks are learning similar features, and the deviations seen are likely due to the mathematical randomness of neural network optimisation via steepest descent and the finite hyperparameter space tested, rather than anything inherent about the individual networks. ResNet50 will be used as the baseline for the remainder of this paper due to its slightly better performance in step classification, and to remain consistent with the most commonly used baseline in workflow recognition.

### Spatial-temporal recognition

To improve on the purely spatial recognition methods, state-of-the-art automated workflow recognition spatial-temporal methods [3, 5, 6, 9] were also tested. For the neural network methods, the optimiser used is SGD with learning rate 0.001 and momentum 0.9, and the Max activation function is used for the classification layer. All images are colour normalised to the ImageNet distribution and resized to 224$\times$224 pixels. The images are then converted into clips of consecutive images, corresponding to the equivalent number of seconds within an operation. To prevent class imbalance, all classes in the training datasets were downsampled to the class with the fewest number of clips. The training is not end-to-end in order to have a fair comparison between the improvements of each respective temporal method.

The first temporal method is a recurrent network, utilising the PyTorch LSTM, with a 512 hidden size as found in SingleNet [5], which achieved an 86.4% accuracy on cholecystectomy. The classification layer of the baseline ResNet50 is removed and the 2048 feature output is fed into the LSTM with a clip size of both 5 and 10 (for two distinct LSTMs) before final classification. The second method is a TCN, utilising TeCNO as presented in [6] with no architecture alterations, where an 88.6% accuracy was achieved on cholecystectomy. In this case, the baseline is a two-headed ResNet50, which contains an additional identity layer before the final layer output (although trained identically to the normal ResNet50). The 2048 features from the feature layer are fed into a 2-stage TCN with a clip size of 30 before final classification.    The final temporal method is a HMM, which uses the output predictions of ResNet50 as an input sequence, rather than the output features as utilised in the neural network methods. Based off the Python library hmmlearn, a Gaussian HMM is used for phases and a Multinomial HMM is used for steps. Using the Viterbi algorithm, completed sequences were used for training, and for validation knowledge of all previous states plus the next five were used. In practice, this would mean the HMM would have an acceptable 3 second delay.

**Fig. 4:**
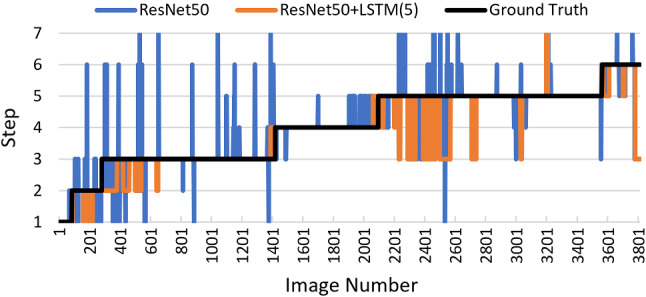
A time-series display of ResNet50 and ResNet50+LSTM(5secs) predictions on a validation video, compared to the ground truth. The spikes and blocks show the rapid change in predictions, moving away from the correct class for a few images before returning back to the correct class

**Fig. 5:**
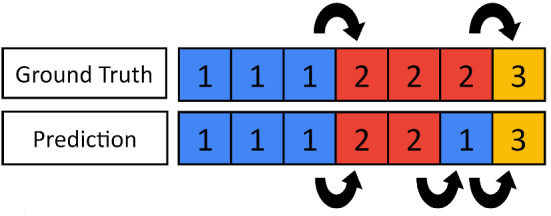
An example of how prediction volatility is calculated from a given ground truth sequence and prediction sequence. In the prediction sequence there are 3 class changes, whereas in the ground truth sequence there are 2 class changes. Therefore, the prediction volatility in this example case is $3/2=1.5$

### Temporal smoothing

Motivated by the short-term prediction volatility of the ANN predictions as displayed in Fig. 4, it was thought TSFs could improve performance while reducing prediction volatility. This new evaluation metric is formally defined as the ratio between the total number of times a class changes in the prediction and the total number of times the class changes in the ground truth, an example is displayed in Fig. 5. Two simple yet novel TSFs were created.

**Fig. 6:**
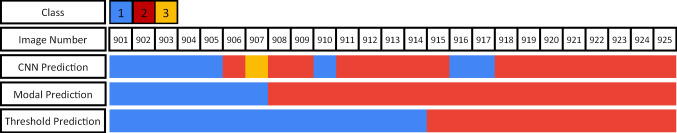
$M_5$ and $T_5$ acting on example CNN predictions. The transition between class 1 and class 2 is more clearly identifiable, and the total number of class changes is reduced from 7 to 1 in both cases, which would the prediction volatility from 7 to 1

The first TSF is a modal function ($M_n$) which takes the modal prediction of the current and previous *n* images, and outputs this as the new prediction. (The first *n* images are unchanged from the prediction, and if there are two modal values the most recent value is used as the prediction.) This is thought to reduce prediction volatility and improve performance as the majority of the predictions are correct, and hence outnumber any incorrect predictions, particularly if there are several incorrect prediction classes as each one would individually only have a few images.

The second is a threshold function ($T_n$) which ensures the predicted class of the current and previous *n* images are the same, and if not, the predicted class is unchanged. (The first *n* images are unchanged from the prediction.) This is thought to reduce prediction volatility and improve performance as a few incorrect predictions will not cause the threshold prediction to change, and the threshold prediction will only change after a period of consistent prediction.

The two functions differ in one major way; in the case of rapid and repeated changes between two predicted classes. The modal function may (correctly) switch to the new class once the new class outnumbers the previous class, but the threshold function will keep to the current class until there is a longer sequence where the new class is predicted. Figure 6 shows an example of both TSFs independently acting on the predictions of a baseline CNN, displaying the reduced prediction volatility and differences. For training, *n* is varied incrementally between [1, 60], and the value that optimises the weighted-$F_1$ score on the training dataset is chosen and applied to the validation dataset.

**Table 1:** Performance of the various spatial methods, maximising online weighted-$F_1$ score. Values are given to 3 significant figures to distinguish between similar CNN performances. A bolded value indicates the best performing model on that column’s evaluation metric

	CNN	Weighted-	Mean-	Weighted-	Weighted-
		$F_1$ score	accuracy	precision	recall
Phases	AlexNet	0.753±0.02	0.744±0.31	0.769±0.30	0.755±0.25
	ResNet34	0.798±0.05	0.794±0.06	0.804±0.04	0.803±0.03
	ResNet50	0.803±0.04	0.795±0.04	**0.811**±**0.04**	0.802±0.04
	ResNet101	0.799±0.05	0.796±0.05	0.808±0.04	0.804±0.05
	DenseNet121	0.800±0.03	**0.838**±**0.04**	0.804±0.04	0.806±0.03
	DenseNet161	**0.804**±**0.03**	0.799±0.05	0.795±0.04	0.802±0.05
	DenseNet201	0.795±0.05	0.809±0.04	0.802±0.05	0.804±0.04
	EfficientNetB0	0.801±0.03	0.801±0.04	0.799±0.05	**0.811**±**0.04**
	EfficientNetB1	0.793±0.03	0.795±0.04	0.810±0.02	0.805±0.03
Steps	AlexNet	0.572±0.06	0.564±0.05	0.599±0.05	0.577±0.05
	ResNet34	0.657±0.04	0.649±0.04	0.693±0.04	0.669±0.05
	ResNet50	**0.666**±**0.03**	0.655±0.02	0.693±0.04	0.662±0.03
	ResNet101	0.664±0.05	0.648±0.03	**0.700**±**0.04**	0.655±0.05
	DenseNet121	0.663±0.04	0.647±0.04	0.688±0.05	0.660±0.04
	DenseNet161	0.660±0.05	0.649±0.03	0.690±0.03	0.658±0.04
	DenseNet201	0.659±0.04	0.637±0.02	0.692±0.04	0.651±0.04
	EfficientNetB0	0.665±0.04	**0.664**±**0.04**	0.689±0.04	0.676±0.04
	EfficientNetB1	0.661±0.04	0.659±0.04	0.690±0.05	**0.679**±**0.03**

## Results

### Spatial recognition

The results displayed in Table 1 show a basic transfer-learnt CNN can effectively discriminate between the various surgical phases and steps of a surgical video, and from the small standard deviations it can be concluded that this result is consistent across the videos. As previously mentioned, the networks themselves (outside of AlexNet) all perform similarly, with the only major difference being the $\sim$20% shorter runtime from the EfficientNet networks. Additionally, the best performing network is often found within the first few epochs, implying there is some overfitting to the training dataset at later epochs.

**Table 2:** Performance of the spatial-temporal methods compared to the baseline spatial method, maximising online weighted-$F_1$ score. Values are given to 2 significant figures. A bolded value indicates the best performing model on that column’s evaluation metric per classification type

	Method	Weighted-	Mean-	Weighted-	Weighted-
		$F_1$ score	accuracy	precision	recall
Phases	ResNet50	0.80±0.04	0.80±0.04	0.81±0.04	0.80±0.04
	TeCNO	0.81±0.04	0.80±0.04	0.82±0.04	0.81±0.04
	ResNet50+LSTM(5)	0.82±0.03	0.82±0.03	0.83±0.03	0.82±0.03
	ResNet50+LSTM(10)	**0.84**±**0.03**	0.83±0.03	0.85±0.03	**0.84**±**0.03**
	ResNet50+HMM	0.81±0.05	**0.83**±**0.04**	**0.85**±**0.05**	0.81±0.04
Steps	ResNet50	0.67±0.03	0.66±0.02	0.69±0.04	0.66±0.03
	TeCNO	0.68±0.05	0.66±0.03	0.70±0.05	0.68±0.04
	ResNet50+LSTM(5)	0.71±0.04	0.69±0.03	0.73±0.04	0.71±0.04
	ResNet50+LSTM(10)	**0.74**±**0.04**	**0.72**±**0.04**	**0.75**±**0.04**	**0.73**±**0.04**
	ResNet50+HMM	0.67±0.04	0.66±0.04	0.74±0.03	0.67±0.04

**Table 3:** Performance of the temporal smoothing functions, maximising online weighted-$F_1$ score. The top values are for phases, the bottom for steps, given to 2 significant figures, except for predicted volatility which are rounded to the closest integer. A bolded value indicates the best performing temporal smoothing function on that column’s evaluation metric per classification type per underlying method

Method	TSF	Weighted-	Mean-	Weighted-	Weighted-	Prediction
		$F_1$ score	accuracy	precision	recall	volatility
ResNet50	-	0.80±0.04	0.80±0.04	0.81±0.04	0.80±0.04	126±43
	$M_n$	0.83±0.04	0.82±0.04	0.86±0.03	0.83±0.04	13±5
	$T_n$	**0.83**±**0.04**	**0.82**±**0.04**	**0.87**±**0.03**	**0.83**±**0.05**	**7**±**4**
ResNet50	-	0.82±0.03	0.82±0.03	0.83±0.03	0.82±0.03	24±7
+LSTM(5)	$M_n$	0.84±0.03	**0.84**±**0.04**	**0.87**±**0.03**	0.84±0.03	12±5
	$T_n$	**0.84**±**0.03**	0.84±0.04	0.87±0.03	**0.84**±**0.02**	**8**±**2**
ResNet50	-	0.84±0.03	0.83±0.03	0.85±0.03	0.84±0.03	13±3
+LSTM(10)	$M_n$	0.86±0.02	**0.85**±**0.02**	**0.89**±**0.01**	0.86±0.02	9±3
	$T_n$	**0.86**±**0.02**	0.85±0.03	0.89±0.01	**0.87**±**0.02**	**6**±**1**
ResNet50	-	0.67±0.03	0.66±0.02	0.69±0.04	0.66±0.03	107±43
	$M_n$	**0.70**±**0.04**	**0.69**±**0.03**	**0.78**±**0.03**	**0.70**±**0.04**	9±5
	$T_n$	0.70±0.05	0.69±0.03	0.76±0.04	0.70±0.05	**6**±**1**
ResNet50	-	0.71±0.04	0.69±0.03	0.73±0.04	0.71±0.04	19±3
+LSTM(5)	$M_n$	0.73±0.05	0.72±0.04	**0.78**±**0.04**	0.73±0.04	10±3
	$T_n$	**0.73**±**0.05**	**0.72**±**0.05**	0.78±0.05	**0.74**±**0.04**	**4**±**1**
ResNet50	-	0.74±0.04	0.72±0.04	0.75±0.04	0.73±0.04	10±1
+LSTM(10)	$M_n$	0.74±0.05	**0.73**±**0.04**	**0.80**±**0.05**	**0.75**±**0.04**	9±3
	$T_n$	**0.75**±**0.05**	0.73±0.04	0.79±0.05	0.75±0.04	**4**±**1**

### Spatial-temporal recognition

**Fig. 7:**
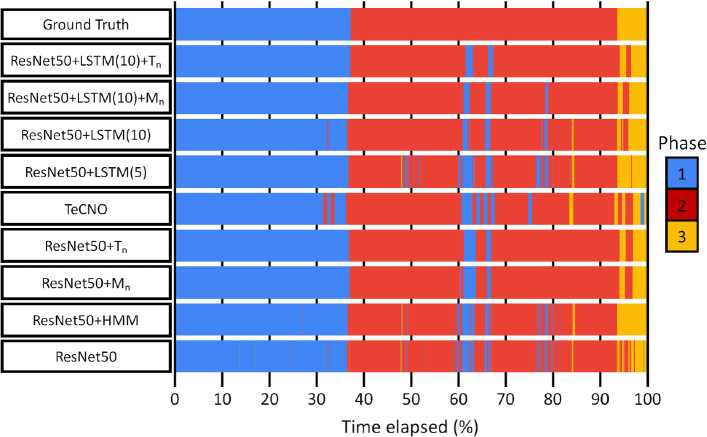
A time-series display of 3-phase classification on a single validation video, comparing predictions from various methods to the ground truth

As displayed in Table 2, ResNet50+LSTM with a 10 second window outperforms the other methods, with a 0.034 and 0.069 weighted-$F_1$ score improvement over the baseline ResNet50 for phases and steps, respectively. Although the 5 second window LSTM outperforms the baseline, it is outperformed by the 10 second window, which is consistent with observations in other surgical analysis tasks [5]. It is also found that TeCNO obtains the highest validation weighted-$F_1$ score when the training weighted-$F_1$ score is the lowest in the first epoch, implying the temporal features learnt by the TCN are not generalisable. Although the weighted-$F_1$ score improvement from using an HMM is small, the HMM does have the best mean-accuracy improvement for phases, and an example of this improvement is displayed in Fig. 7. Here, the HMM accurately predicts the phase 2 to phase 3 transition, due to the transition matrix learning that once a prediction is in phase 3 it is very unlikely to change.

### Temporal smoothing

From Table 3 it can be seen that the two TSFs consistently increase the weighted-$F_1$ score while significantly reducing the prediction volatility. Focusing on the baseline ResNet50 improvement, it is found both modal and threshold smoothing improve performance to values comparable to the ResNet50+LSTM(5). This also means the simple TSFs outperform the more sophisticated TCN and HMM methods, which is interesting to note. Moreover, when applied on top of the ResNet50+LSTM predictions, improvement comparable to the initial LSTM improvement is seen. For instance, for phases, ResNet50+LSTM(10) improves the weighted-$F_1$ score by 0.033 when compared to the baseline, and is further improved by 0.027 once threshold smoothing is applied; an example of this is displayed in Fig. 7.

**Fig. 8:**
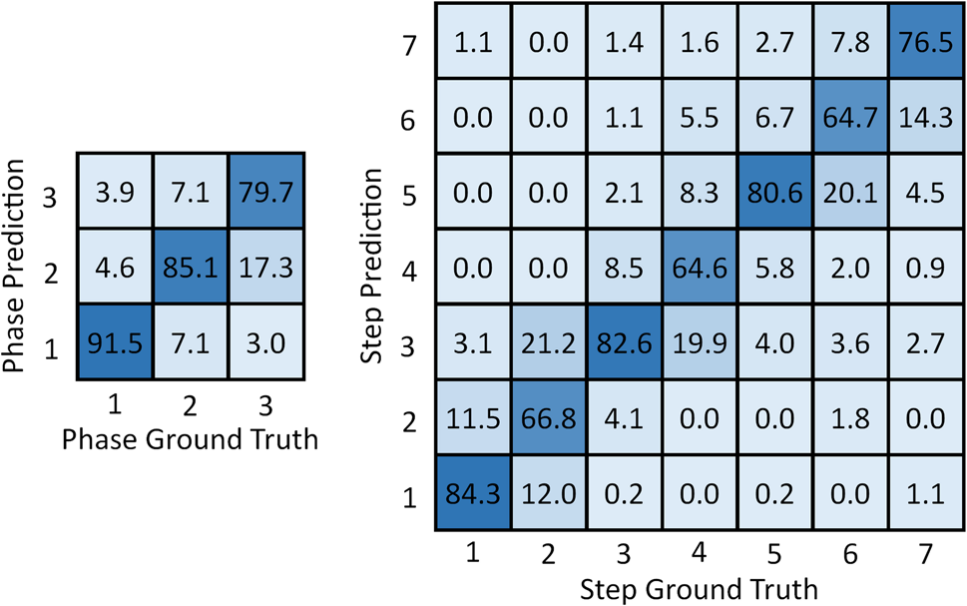
Multi-class confusion matrix for optimal method, ResNet50+LSTM(10) with threshold smoothing, on 3-phase and 7-step classification. Values are averaged across all 5 cross-validation folds and given as percentages to 1 decimal place. The values on the diagonal represent the precision for each class, and the remaining values represent the false discovery rate

Comparing the TSFs against each other, in general, threshold smoothing outperforms modal smoothing, while also reducing the prediction volatility to a greater extent. Both differences are due to the aforementioned functional difference when smoothing out rapidly changing predictions. This is highlighted in Fig. 7, looking at the TSFs acting on ResNet50+LSTM(10) predictions; the modal function incorrectly switches from phase 2 to phase 1 at $\sim$77% time elapsed, whereas the threshold function remains the same. Figure 8 shows a confusion matrix for the optimal method. From this, it can be seen that images are frequently misclassified to classes neighbouring to the ground truth class, but otherwise not misclassified. For example, step 2 is misclassified as step 3 21.2% of the time, but never misclassified as step 4. Interestingly, step 1 is sometimes misclassified as step 7 but never as step 4, 5, or 6. This is perhaps due to the entry images in step 1 being similar to the exit images of step 7.

## Conclusion

In this paper, a detailed comparison of the established automated surgical workflow recognition methods has been applied to 50 videos of endoscopic pituitary surgery, demonstrating their effectiveness on a new dataset of a surgical approach that utilises a small working space. Weighted-$F_1$ score was used as the primary evaluation metric for all method comparisons. For purely spatial recognition, no significant difference was found between the current best performing ImageNet convolution neural networks. Knowing this, ResNet50 was chosen as the baseline network as to be consistent with other automated operative workflow recognition tasks. For spatial-temporal recognition, it was shown long short-term memory networks with a 10 second window outperformed long short-term memory networks with a smaller 5 second window, as well as temporal convolution networks and hidden Markov models.

Although these methods were able to account for variation in step sequencing, they were unable to account for uninformative images caused by the frequent scene occlusions and endoscope withdrawals. This results in rapidly changing predictions, and so a new evaluation metric, prediction volatility, was created in order to measure these phenomena and act as a proxy for uncertainty. To reduce the prediction volatility, two simple temporal smoothing functions were created and applied to the predictions of the spatial-temporal methods. These functions were shown to not only reduce prediction volatility, but also improve the weighted-$F_1$ score, with the threshold smoothing function being more effective at both evaluation metrics when compared to the modal smoothing function. Additionally, when applied to the baseline spatial predictions, in many cases the smoothing functions outperform the established spatial-temporal methods. Hence, temporal smoothing functions are effective at reducing prediction volatility, which in turn lowers the model’s uncertainty and increases a clinician’s confidence in using artificial neural networks for surgical workflow recognition.
